# Flies

**Published:** 1901-07

**Authors:** 


### EDITORIAL NOTES AND COMMENTS.

The Business office of THE JOURNAL-RECORD is No. 64 Marietta Street.

The Editorial office is 318-319-320 The Prudential.

Address all Business communications to Dr. M. B. Hutchins, Mgr.

Make remittances payable to THE ATLANTA JOURNAL-RECORD OF MEDICINE.

On matters pertaining to the Editorial and Original Communications address Dr. Bernard Wolff, Atlanta.

Reprints of original articles will be furnished at cost price. Requests for the same should always be made *on the manuscript*.

We will present, post-paid, on request, to each contributor of an original article, twenty (20) marked copies of THE JOURNAL-RECORD containing such article.

### FLIES.

THIS is preeminently the fly season. They may be seen, heard and felt at all times during the day and at all places where there is sufficient warmth to keep them alive. If one were so unfortunate as to be able to follow the fly throughout its daily wanderings, he would probably desire to spend the remainder of the summer in the seclusion offered by a cold storage vault. The discouragements in the matter of eating would be sufficient to cast a dense shadow over the joys of the table. But luckily all flies look alike, and we have no ready means of identifying the languid struggler in the soft butter with the one we encountered earlier in the day in the bureau of terminal alimentary distribution. We resolutely set our faces against the possibility that the resemblance may be more than family or casual, and that we have met before under different and somewhat less embarrassing circumstances. We confine ourselves to the thing *in esse* and help ourselves from that portion of the butter which lies most remotely from the arena of the death struggle and less likely to have been the *point d'appui* of the earlier and more vigorous evolutions to effect disentanglement and escape, without giving rein to conjecture or theory upon the undiscriminating and complaisant dietary tastes of the condemned. An adroit flick liberates the table of the obnoxious presence, and, save for a consciousness of diminished acuteness of appetite, the episode is closed. But bacteriology has of late years added an element of fear to our wholesome disgust of the fly. It has been demonstrated that these winged pests often become the intermediary host of the micro-organisms of communicable disease, of typhoid fever in particular, and that preventive sanitation should include efforts at elimination of these vehicles of transmission. It will be remembered that the prevalence of typhoid at Chickamauga during the late war with Spain was attributed in large measure to the plague of flies. It is more than likely that many other diseases may be spread by this means, and instead of regarding this insect as one of nature's disinfectors as some utilitarians do, it should be looked upon as a winged messenger of evil, and the black flag raised against it. Bacteriology is hardly needed to convince one of the truth of this notion of flies' malevolent mission. Their fondness for filth and foul matters is a conspicuous trait in their character. How greedy and horrible they are about a discharging wound, and with what pertinacity they return when frightened away from their revolting feast! It requires very little play of imagination to supply the hiatus in the rationale of dissemination of disease by this means. If the fly has any place in nature for good it must lie in the amusement which it affords to the small boy as an object of pursuit and capture in the interest of sport. How many tedious hours of parentally enforced attendance upon church have been pleasantly diversified by the fascinating pastime of catching flies. A sly and hurried sweep of the half-opened hand along the surface of the clothing or the back of the pew in front, the careful extraction of the victim from between the fingers, the amputation of the oviduct and the insertion into the aperture of a streamer of paper torn from the appropriately named fly-leaf of the hymn book, is the work of a short and busy minute. Then the delirious but repressed delight of watching the vagrant flight of the pennanted insect over the heads of the unconscious congregation until the eye can follow it no longer in the dusky corner under the choir's gallery. Then for repetition of the sport, which becomes so absorbing that the ear scarcely recognizes the slap of the Bible closing and the words long awaited and radiant with promise, "Now, in conclusion, brethren."

These are among the green spots of childish memory, and serve to throw a glamor of affection around the fly which subsequent experience does not entirely disperse. Besides the small boys' efforts at destruction, willing but of limited effectiveness, the means of exterminating the pests are very crude and primitive. Woven-wire screens and tangle-foot paper, as weapons of defense and offense, constitute the entire arsenal at present. The man who will invent something more scientific and more efficient will not only be a benefactor of his race but will find a pot of gold waiting for him. Why does not some inventor like Maxim or Nordenfeldt turn his attention to devising implements of war to be used against a mimic but formidable enemy of mankind?

